# Joint Bodies, from within and without, in Articulations Otherwise Normal

**Published:** 1915-05

**Authors:** 


**Joint Bodies, From Within and Without, in Articulations Otherwise Normal.** Aime Paul Heineck, Ill. Med. Jour., Jan. 1915, discusses this subject systematically. The article does not lend itself to abstracting but is valuable for reference.

